# Lentiviral vector gene therapy and CFTR modulators show comparable effectiveness in cystic fibrosis rat airway models

**DOI:** 10.1038/s41434-024-00480-y

**Published:** 2024-08-25

**Authors:** Alexandra McCarron, Kak-Ming Ling, Samuel T. Montgomery, Kelly M. Martinovich, Patricia Cmielewski, Nathan Rout-Pitt, Anthony Kicic, David Parsons, Martin Donnelley

**Affiliations:** 1 Adelaide Medical School, The University of Adelaide, Adelaide, SA Australia; 2 Robinson Research Institute, The University of Adelaide, Adelaide, SA Australia; 3 Department of Respiratory and Sleep Medicine, Women’s and Children’s Hospital, North Adelaide, SA Australia; 4 Wal-Yan Respiratory Research Centre, Telethon Kids Institute, Nedlands, WA Australia; 5 School of Population Health, Curtin University, Bentley, WA Australia; 6 Centre for Child Health Research, University of Western Australia, Crawley, WA Australia; 7 Department of Respiratory and Sleep, Perth Children’s Hospital, Nedlands, WA Australia; 8 Centre for Cell Therapy and Regenerative Medicine, School of Medicine and Pharmacology, The University of Western Australia and Harry Perkins Institute of Medical Research, Nedlands, WA Australia

**Keywords:** Respiratory tract diseases, Genetic vectors

## Abstract

Mutation-agnostic treatments such as airway gene therapy have the potential to treat any individual with cystic fibrosis (CF), irrespective of their CF transmembrane conductance regulator (*CFTR*) gene variants. The aim of this study was to employ two CF rat models, Phe508del and *CFTR* knockout (KO), to assess the comparative effectiveness of CFTR modulators and lentiviral (LV) vector-mediated gene therapy. Cells were isolated from the tracheas of rats and used to establish air-liquid interface (ALI) cultures. Phe508del rat ALIs were treated with the modulator combination, elexacaftor-tezacaftor-ivacaftor (ETI), and separate groups of Phe508del and KO tracheal epithelial cells were treated with LV-CFTR followed by differentiation at ALI. Ussing chamber measurements were performed to assess CFTR function. ETI-treated Phe508del ALI cultures demonstrated CFTR function that was 59% of wild-type level, while gene-addition therapy restored Phe508del to 68% and KO to 47% of wild-type level, respectively. Our findings show that rat Phe508del-CFTR protein can be successfully rescued with ETI treatment, and that *CFTR* gene-addition therapy provides significant CFTR correction in Phe508del and KO ALI cultures to levels that were comparable to ETI. These findings highlight the potential of an LV vector-based gene therapy for the treatment of CF lung disease.

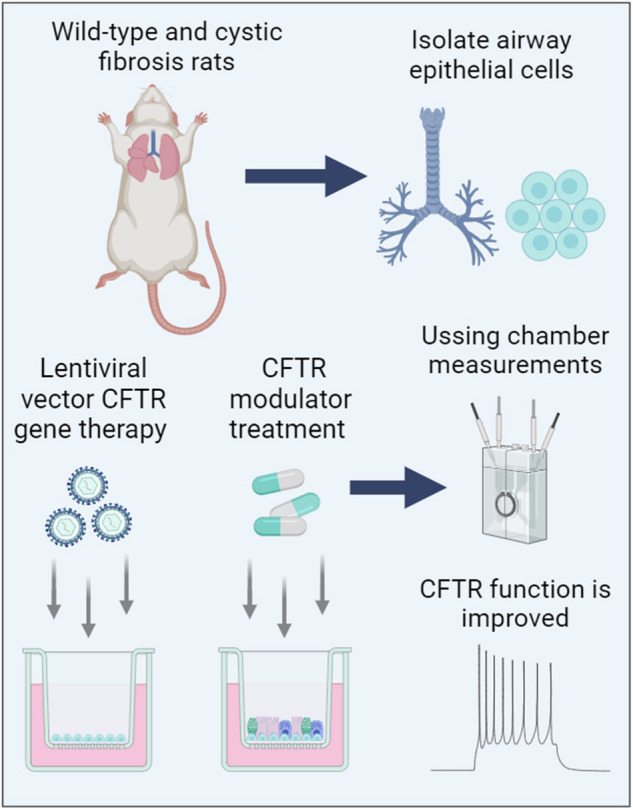

## Introduction

Cystic fibrosis (CF) is a genetic disorder that arises from pathogenic variants in the CF transmembrane conductance regulator (*CFTR*) gene. The *CFTR* gene encodes for an ion channel that is responsible for the transport of chloride and bicarbonate ions in epithelial cells. In CF, this ion channel is dysfunctional, leading to disease manifestations in a range of organs, including the lungs. Pulmonary disease is characterised by an imbalance in ion and water transport, subsequent dehydration of the airway surface liquid, and increased viscosity of the overlying mucus. Mucus-stasis creates an environment that is highly susceptible to colonisation by microbes. Over time, persistent infections produce a state of chronic inflammation, leading to tissue damage and impaired lung function [1].

Research is ongoing to further elucidate the mechanisms underlying CF lung disease pathogenesis and to develop effective treatment strategies. Prior to human assessment and to facilitate these investigations, genetically modified animals with defects in the *CFTR* gene have been generated to model CF disease including mice [2], rats [3–6], ferrets [7] and pigs [8], each having their own benefits and limitations [9, 10]. We have generated and characterised two CF rat models, one with a *CFTR* knockout (KO; Class I) and another with the common Phe508del (Class II) mutation. These rat models demonstrate CF phenotypes including intestinal obstruction, malformation of the male reproductive tract, and electrophysiological defects in the nasal epithelium [5]. However, they do not demonstrate histopathological evidence of lung disease. Moreover, it is currently unknown whether the CF electrophysiological defects observed in the nasal epithelium are replicated in the trachea, and if so, whether treatment with available modulator drugs can restore CFTR function.

CFTR modulators are a highly effective therapy for most people with CF. However, thousands of individuals are unable to benefit from these medications for a range of reasons including having refractory *CFTR* variants that are not amenable to pharmacological rescue (~10%), poor tolerability due to side effects, minimal clinical benefit despite having suitable variants, or lack affordable access due to location [11]. Modulators are small molecule compounds that act to either increase the amount of mature CFTR protein that is trafficked to the cell surface (corrector) or restore ion conductance by increasing opening of the CFTR channel (potentiator) [11]. Elexacaftor-tezacaftor-ivacaftor (ETI) is a triple combination modulator therapy consisting of two corrector molecules and a potentiator. Treatment with this modulator combination has proven to be highly effective in patients with at least one copy of the Phe508del variant and has resulted in improved lung function and reduced frequency of pulmonary exacerbations [12, 13]. As CFTR modulators are now considered the gold standard treatment for most people with CF, all new therapies will likely need to demonstrate comparable effectiveness in their ability to restore CFTR function.

Alternative therapeutic strategies are under development for individuals that cannot receive modulator therapies, including genetic-based therapies that target the airway epithelium. Airway gene-addition therapy is one approach that involves delivering wild-type copies of the *CFTR* gene to the airway cells, with the intention of restoring CFTR ion channel function. Viral vectors such as adeno-associated virus (AAV) vectors and lentiviral (LV) vectors are considered the leading candidates for CF airway gene therapy, and their advantages and disadvantages have been reviewed in detail previously [14–16]. Using an LV vector approach, we have demonstrated restoration of CFTR function in the nasal epithelium of CF mice and CF rats [17, 18], while others have shown promising results using LV vectors to restore CFTR activity in human CF cell culture systems [19, 20] and the lower airways of CF pigs [21]. While we have shown correction in the upper nasal airways, we need to examine gene-correction in the lower airways, which more accurately recapacitate the target for CF gene therapy. The functional correction levels that can be achieved with gene-addition therapy in comparison to CFTR modulators also remains unknown.

The aim of this study was to characterise CFTR function in air-liquid interface (ALI) cultures derived from the trachea of Phe508del and KO rats, and to determine if Phe508del rat airway cells are responsive to ETI. Following this, we sought to compare CFTR restoration levels following LV vector gene-addition therapy or ETI.

## Materials and methods

### Animals

This study was approved by the University of Adelaide animal ethics committee under application M-2019-038 and was conducted in accordance with ARRIVE guidelines. Male and female wild-type, Phe508del and *CFTR* knockout rats >8 week of age were employed. CF rats were maintained using previously described husbandry measures [5].

### Rat tracheal epithelial cell isolation and air-liquid interface (ALI) culture

Rats were humanely killed by CO_2_ asphyxiation. Tracheal epithelial cells were isolated by pronase dissociation as previously described [22]. Briefly, tracheas were cannulated, excised, and infused with 1% pronase (Roche, Switzerland) solution in Ham’s F12 media (Sigma-Aldrich, MO, USA) supplemented with 1% PenStrep, 0.05 mg/mL Gentamicin, and 0.25 µg/mL Amphotericin B. Tracheas were incubated for 18-24 hours at 4 °C. On day two, epithelial cells were retrieved by flushing the tracheas with 30 mL Ham’s F12 media. Cells were collected by centrifugation (500 *x g*, 4 °C, and 10 mins) and incubated on ice with 0.5 mg/mL DNase (Sigma-Aldrich) and 10 mg/mL bovine serum albumin (BSA) (Sigma-Aldrich) to reduce cell clumping. Cells were spun down, resuspended in PneumaCult Ex-Plus media (#5040, StemCell Technologies, Canada) and assessed for number and viability. Cells were seeded directly onto 6-well Snapwell inserts (#CLS3407, Corning, NY, USA) pre-coated with 1:100 rat-tail collagen type I in phosphate-buffered saline (PBS) at a density of 300,000 cells per well. Once confluent, the growth media was removed from both apical and basolateral compartments and the basolateral compartment only was replaced with PneumaCult ALI media (#5001, StemCell Technologies). Media in the basolateral chamber was refreshed every 48 hours.

### Immunohistochemistry (IHC)

Inserts were fixed in 10% neutral buffered formalin for 15 minutes, embedded in paraffin wax, and sectioned at 5 μm. Sections were deparaffinised and stained with hematoxylin and eosin (H&E), or processed for IHC as follows. Heat-mediated antigen retrieval was performed in sodium citrate buffer (pH 6.0) for 20 minutes. Sections were blocked with 1% BSA and 0.05% Tween-20 for one hour at room temperature. Primary antibodies were prepared in a dilution buffer consisting of 1% BSA and 0.1% Triton X-100 in PBS. Antibodies that were used included acetyl-alpha tubulin (5335, Cell Signalling Technology, MA, USA) at 1:500 dilution, cytokeratin 5 (ab52635, Abcam, United Kingdom) at 1:400 dilution and MUC5AC (MA5-12178, Invitrogen, MA, USA) at 1:200 dilution. Sections were incubated with primary antibodies overnight at 4 °C. Slides were washed thoroughly and incubated at room temperature for one hour with secondary antibodies donkey anti-rabbit (IgG) Alexa Fluor 488 (ab150073, Abcam) or goat anti-mouse (IgG) Alexa Fluor 568 (A-11004, Invitrogen) at a 1:200 dilution in antibody dilution buffer. The secondary antibody was washed and a DAPI nuclear counterstain was applied (4083S, Cell Signalling Technology). Slides were washed and mounted with ProLong Diamond Antifade mountant (P36970,  Invitrogen). Bright field images were captured on a Nikon Eclipse E400 microscope and fluorescence images on a Nikon Eclipse Ts2 with DS-Fi3 camera and NIS-elements D software version 4.20.02.

### Transepithelial electrical resistance (TEER) measurements

At 7-10 days post air-lift, multilayered cells, mucus and cilia were all visible under light microscopy suggesting terminal differentiation of cultures. TEER measurements were then performed to ensure the appropriate formation of cell junctions and cell integrity. Using an epithelial voltammeter (Millicell-ERS voltmeter, Millipore) with silver chloride “chopstick” electrodes, triplicate measurements of TEER were obtained for each well followed by Ussing chamber measurements. Resistance obtained from a cell-free culture was subtracted from that measured across each culture and corrected for surface areas of inserts (1.1 cm^2^) to yield the TEER of the epithelial cells with values expressed in Ω/cm^2^.

### Short-circuit current (I_sc_) measurements

Baseline CFTR function was assessed in wild-type (*n* = 14), Phe508del (*n* = 13) and KO (*n* = 12) rat ALI cultures that were generated from individual animals. At 7–10 days post air-lift the transepithelial short-circuit current (I_sc_) was measured using an Ussing chamber (VCC MC6, Physiologic Instruments, NV, USA) as previously described [23]. Cells grown on Snapwell inserts were mounted into sliders (P2302, Physiologic Instruments) in the Ussing chamber and submerged in Krebs Ringer Buffer (KRB) that was bubbled with carbogen (95% O_2_, 5% CO_2_). After compensating for voltage offsets, the transepithelial voltage was clamped at 0 mV and current and resistance were recorded with the Acquire and Analyze software (Physiologic Instruments). To assess chloride ion transport, sodium transport was blocked with the addition of amiloride bilaterally (10 µM final concentration) before stimulation of cAMP-mediated chloride transport with the addition of forskolin (10 µM final concentration) and blocking of CFTR transport using CFTR_inh_-172 (30 µM final concentration). Appropriate periods of equilibration between each addition were used. To calculate changes in I_sc_, the average current measured over the 60 seconds following amiloride addition was subtracted from the average current after forskolin measurement.

### CFTR modulator treatment of rat ALI cultures

Differentiated rat ALI cultures generated from individual animals (*n* = 8) were treated via the basolateral chamber with modulators 24 hours prior to short-circuit current measurements. Cells were treated with 3 µM elexacaftor (VX-445), 18 µM tezacaftor (VX-661), and 1 µM ivacaftor (VX-770) (Selleck Chemicals, TX, USA) as previously described [24]. A DMSO vehicle control was included with no differences observed to the untreated controls (data not shown). Following treatment, TEER and short-circuit current measurements were performed as described above.

### LV-GFP vector transduction of rat airway epithelial cells

The VSV-G (vesicular stomatitis virus glycoprotein) pseudotyped EF1α-3XFLAG-fLuc-eGFP LV vector was produced in-house and was titered using flow cytometric detection of green fluorescent protein (GFP) as previously described [25]. Wild-type rat airway epithelial cells were seeded into collagen-coated 6-well plates (#152034, Thermo Fisher Scientific, MA, USA) at 25,000 cells per well and were maintained in F-medium containing ROCK inhibitor as previously described [23]. Following adhesion, cells were transduced at a multiplicity of infection (MOI) of 1, 10 or 100 with the appropriate volume of EF1α-3XFLAG-fLuc-eGFP vector at a titre of 1 ×10^9^ transducing units (TU)/mL. Each MOI was tested in triplicate over two separate experiments (*n* = 6 per group in total). The following day, the media was refreshed. Three days post-transduction cells were imaged via fluorescent microscopy (Nikon Eclipse Ts2). Cells were harvested for flow cytometry to determine the proportion of GFP positive cells and to confirm their identity as basal cells. Cells were fixed in 4% paraformaldehyde, permeabilised in 0.1% saponin and stained with anti-cytokeratin 5 antibody (ab52635, Abcam) at a 1:100 dilution for 30 minutes. Cells were washed and stained with Goat anti-rabbit IgG Alexa Fluor 568 (A-11011, Invitrogen) at 1:500 dilution for 30 minutes. Cells were washed and stored overnight at 4 °C in PBS. Samples were measured using an LSRFortessa (BD biosciences, NJ, USA) and analysed using FlowJo software (v10).

### LV-CFTR vector treatment of CF rat airway epithelial cells

The VSV-G pseudotyped EF1α-V5-CFTR LV vector was produced by the Functional Genomics South Australia Core Facility (FGSA) at the University of Adelaide. Vector titration was performed by transducing HEK 293T cells and detecting the V5 epitope tag by flow cytometry, as previously described [18]. The titre of the vector employed was 1.5 ×10^8^ TU/mL. Tracheal cells were isolated from Phe508del (*n* = 5) and KO rats (*n* = 5) and were seeded onto inserts at a density of 300,000 cells per well. After 24 hours, reference wells were used to determine the average number of cells for calculating the MOI. An appropriate volume of LV vector was applied to each well to achieve an MOI of 1, and cells were incubated for approximately 24 hours. The following day the media was replaced, and the cells were airlifted. Following differentiation, TEER and short-circuit current measurements were performed as described above.

### *CFTR* gene expression analyses by qPCR

Differentiated ALI cultures were lysed in PureLink lysis buffer (Thermo Fisher Scientific) and total RNA was extracted using the RNeasy® kit (Qiagen, Germany) following the manufacturer’s instructions. The quantification and evaluation of the purity of RNA samples was assessed using the NanoDrop™ Lite spectrophotometer (Thermo Fisher Scientific). Reverse transcription of RNA to synthesise complementary DNA was performed using the QuantiTect Reverse Transcription Kit (Qiagen) following the manufacturer’s guidelines. qPCR was performed using the Fast SYBR™ Green Master Mix (Thermo Fisher Scientific) following the manufacturer’s instructions, in line with the Minimum Information for Publication of Quantitative Real-Time PCR Experiments (MIQE) guidelines [26]. Primer sequences are included in the supplementary information (Table S1). The ability of the primers to specifically amplify either rat or human *CFTR* is shown in the supplementary figures (Figures S1 and S2). PCR samples were heated for 10 minutes at 50 °C followed by 3 minutes at 95 °C, then qPCR reactions were run for 40 cycles of 95 °C for 10 seconds (denaturation) and 56 °C or 60 °C for 60 seconds (combined annealing/extension) using a CFX Connect Real-Time PCR Detection System (Bio-Rad, CA, USA). Samples were run alongside a standard curve of known copy numbers for *Cyclophilin A* (*CycA*), rat *CFTR* and human LV-derived *CFTR*. The calculated copy number for rat *CFTR* and human *CFTR* was normalised to *CycA* copy number. PCR products were assessed by gel electrophoresis using a 1% agarose gel with a 100 bp DNA ladder (AXYM-DNA-100bp, Axygen, CA, USA) (Figure S3).

### Statistics

Data was analysed using GraphPad Prism (v9). Where appropriate, data were assessed for normality using the Shapiro-Wilk test, and the Brown-Forsythe and Bartlett’s tests was used determine equal variances between groups. Data were analysed for statistically significant differences using one-way ANOVA with Tukey’s multiple comparisons test. A *p*-value of <0.05 was considered statistically significant. Graphs show the mean ± standard error of the mean (SEM) and reported in-text values are the mean ± standard deviation (SD).

## Results

### ALI cultures can be successfully generated from CF rat trachea cells

**Fig. 1:**
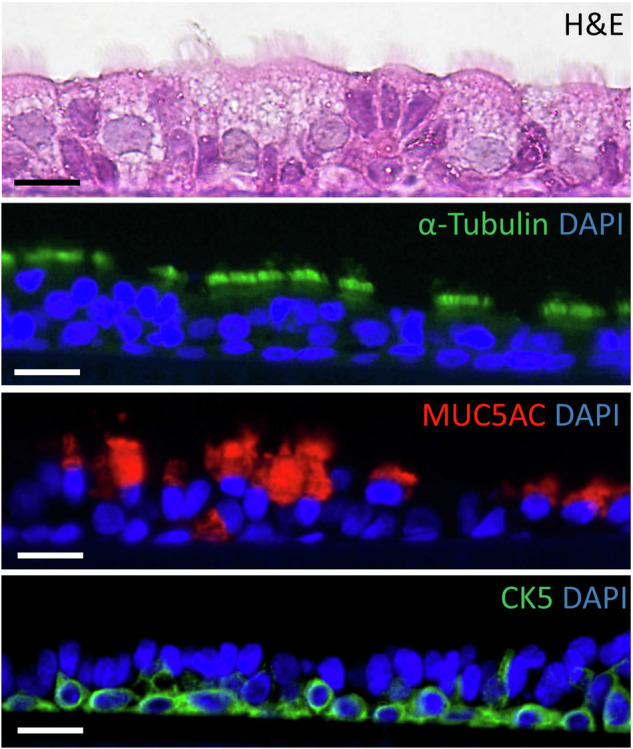
Rat ALI cultures demonstrate mucociliary differentiation. Cellular markers of differentiated cell types identified via immunohistochemistry, including α-tubulin for ciliated cells, MUC5AC for goblet cells, and CK5 for basal cells. Representative images are from a wild-type rat ALI culture at 15-days post air-lift. Scale bar = 10 µM.

Rat-derived ALI cultures from all genotypes developed into a pseudostratified epithelium (Fig. 1). Additionally, the presence of mucus and cilia-beating was observed upon light microscopy, and TEER measurements (data not shown) confirmed the integrity and permeability of the culture. Immunohistochemical staining identified the presence of respiratory cell types including ciliated (ɑ-tubulin), goblet (MUC5AC), and basal cells (cytokeratin 5), with no differences observed in the cellular composition between the rat genotypes.

### Rat airway basal cells can be successfully transduced with LV vector

**Fig. 2:**
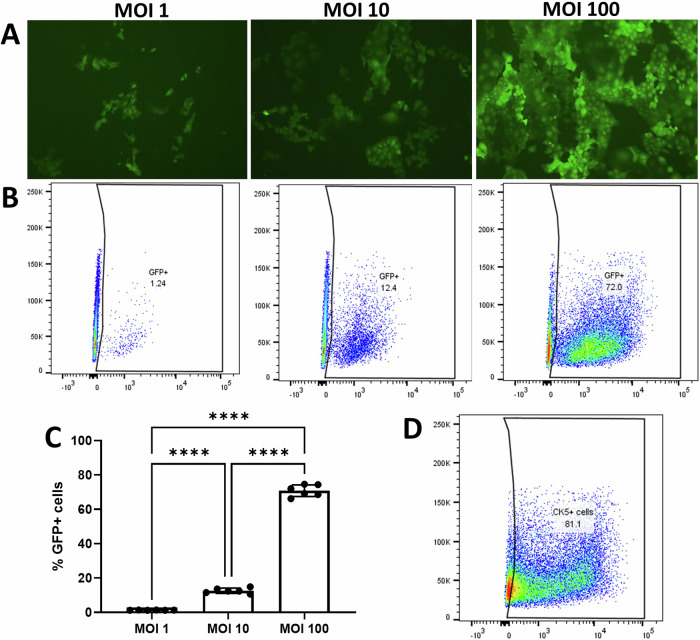
Rat tracheal epithelial cells can be successfully transduced with VSV-G pseudotyped LV vector. **A** Wild-type rat airway cells transduced with LV-GFP at MOIs of 1, 10 and 100 show dose-related increases in the proportion of cells with green fluorescence. **B** Corresponding flow cytometry quantification of the percentage of GFP-positive cells. **C** Graph showing the average percentage of GFP-positive cells at the respective MOIs. **D** Immunostaining with cytokeratin 5 confirmed the identity of the rat airway epithelial cells as predominantly basal. Data are represented as mean ± SEM, one-way ANOVA, Tukey’s multiple comparison test, ****p < 0.0001, *n* = 6 technical replicates per group (performed over two independent experiments).

Rat airway epithelial cell cultures were shown to be permissive to transduction by a VSV-G pseudotyped LV-GFP vector. Flow cytometry performed on the cultured cells confirmed their identity as predominantly basal cells, with 81% staining positive for cytokeratin 5. Cells transduced with the LV-GFP vector at MOI of 1, 10, and 100 showed significant dose-related increases in transduction levels (*p* < 0.0001) (Fig. 2). Flow cytometric quantification of GFP-positive cells showed that an MOI of 1 produced an average of 1.2 ± 0.08% GFP-positive cells, an MOI of 10, 12.6 ± 1.5% and an MOI of 100, 70.8 ± 3.4%.

### Phe508del and KO rats have reduced CFTR function in the tracheal airways

**Fig. 3:**
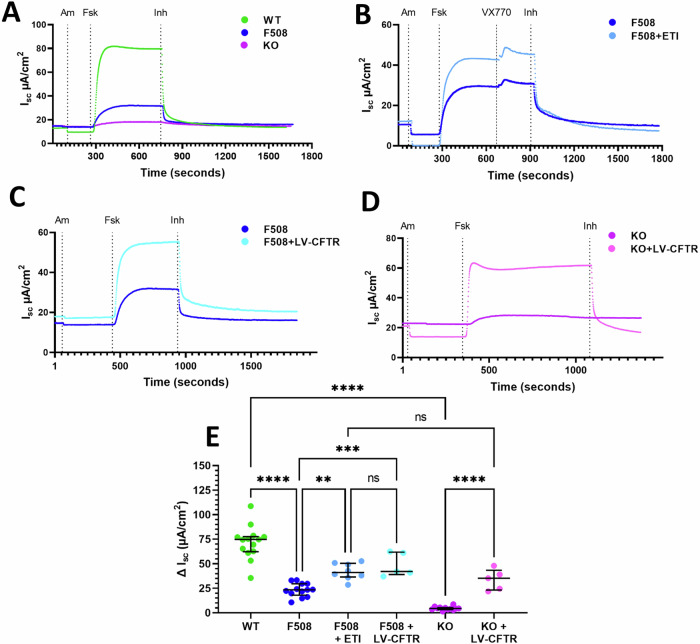
ETI and LV-CFTR treatment show comparable efficacy in restoring CFTR function. Representative I_sc_ traces from **A** ALIs derived from wild-type, Phe508del and KO airway epithelial cells, **B** untreated Phe508del and ETI-treated rat ALI cultures, **C** Phe508del LV-CFTR treated ALI culture compared to untreated Phe508del and **D** KO LV-CFTR treated ALI culture compared to untreated KO. **E** ΔI_sc_ forskolin response in wild-type, Phe508del, KO, Phe508del treated with ETI, and Phe508del and KO treated with LV-CFTR. Data are represented as mean ± SEM, one-way ANOVA, Tukey’s multiple comparison test, ns: not significant, **p < 0.01, ***p < 0.001, ****p < 0.0001, *n* = 5-14 per group. Each data point represents an individual animal. F508; Phe508del.

Using chamber measurements performed on rat ALI cultures revealed a significant reduction in the delta short-circuit current (ΔI_sc_) response to forskolin in both Phe508del (23.25 ± 6.95 µA/cm^2^, *p* < 0.0001) and KO (4.52 ± 1.84 µA/cm^2^, *p* < 0.0001) rats when compared to wild-type (71.66 ± 16.96 µA/cm^2^) (Fig. 3A, E). KO rats had a significantly blunted response to forskolin that was only 6% of wild-type level, while the Phe508del rats had a small response that was 32% of the wild-type, indicating low-level residual CFTR function.

### Phe508del rat airway cells respond to ETI treatment

Rat ALI cultures were treated with the CFTR modulator combination ETI 24 hours prior to assessment. Phe508del ALIs were found to be responsive to modulator treatment indicated by improved CFTR function. Phe508del treated cultures demonstrated a significant increase in the ΔI_sc_ forskolin response (42.17 ± 8.32 µA/cm^2^, *p* = 0.0034) that was corrected to 59% of wild-type level (Fig. 3B, E).

### LV-CFTR gene-addition therapy restores CFTR function in Phe508del and KO rat ALIs

Treatment of CF rat airway epithelial cells with LV-CFTR gene vector at an MOI of 1 produced significant CFTR function upon Ussing chamber measurement of differentiated ALI cultures. Treated Phe508del (48.74 ± 12.03 µA/cm^2^, *p* = 0.0005) and KO (33.72 ± 10.68 µA/cm^2^, *p* < 0.0001) cells exhibited significantly increased ΔI_sc_ forskolin responses when compared to untreated cultures (Fig. 3C–E). On average, the Phe508del rats demonstrated restoration that was 68% of wild-type level, and the KO rats reached 47%. There were no statistically significant differences between the Phe508del ETI-treated group and the Phe508del or KO LV-CFTR treated groups.

### Human *CFTR* transcripts were detected following LV-CFTR treatment

**Fig. 4:**
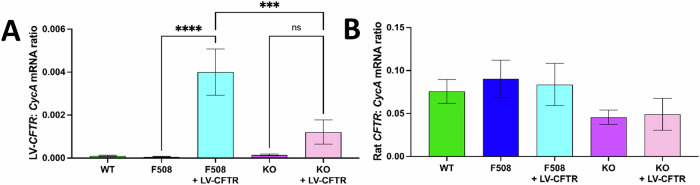
Human *CFTR* mRNA is detected in LV-CFTR treated ALI cultures. Absolute quantification of **A** human and **B** rat *CFTR* mRNA in untreated and LV-CFTR treated rat ALIs. Total copy number is expressed relative to the total copy number of the housekeeping gene *CycA*. Data are represented as mean ± SEM, one-way ANOVA, Tukey’s multiple comparison test, ns: not significant, ***p < 0.001, ****p < 0.0001, *n* = 2–5 per group. F508; Phe508del.

Quantitative PCR (qPCR) showed the presence of human *CFTR* transcripts in Phe508del (*p* < 0.0001) and KO (*p* = 0.078) rat cultures that were treated with LV-CFTR gene-addition therapy, while no h*CFTR* transcripts were detected in the untreated samples (Fig. 4A). The LV-CFTR treated Phe508del cells had significantly higher levels of *hCFTR* mRNA than the treated KO cells (*p* = 0.0004). Treatment with LV-CFTR did not have any effect on the endogenous rat *CFTR* mRNA expression levels (Fig. 4B).

## Discussion

Gene-addition therapy is a promising strategy for tackling CF lung disease and may provide an effective alternative for those without adequate treatment options. Our study demonstrated restoration of CFTR function in ALI cultures derived from Phe508del and KO rat airways, highlighting that a gene-addition approach is effective irrespective of mutation type. This work supports our previous studies that showed improved CFTR function in the nasal airways of CF mice [17, 27] and CF rats [18] following LV-mediated gene therapy when assessed by nasal potential difference (NPD). Here we also revealed that LV gene-addition therapy produces similar levels of CFTR restoration to ETI, an important finding given that modulator drugs are now the benchmark for which all new CF therapies will be compared.

CF rats are proving to be useful models for research investigations. In particular, the development of rat models with human CF-causing variants introduced into the *CFTR* gene such as Phe508del provides the opportunity to test mutation-specific therapies in vivo including CFTR modulators, antisense oligonucleotides, and gene editing strategies [4, 5]. In a separate study, we recently showed that oral treatment of Phe508del rats with ETI produces significant improvements in CFTR function in the nasal airways following NPD assessment [28]. Mouse and rat models have also been generated to express human *CFTR* under control of the endogenous promoter, enabling the gene to be expressed at physiological levels in the appropriate tissues. A rat model expressing human *CFTR* with the Gly551Asp mutation has previously been found to be responsive to ivacaftor with measures including electrophysiology, airway surface and periciliary liquid depth, mucus transport rates, and mucus viscosity showing normalisation following treatment [29].

Our study demonstrated that CF rat trachea-derived ALI cultures could be successfully established, with immunohistochemical staining confirming the presence of cell types including ciliated, basal, and goblet cells. Ussing chamber assessment of CFTR ion channel function revealed that Phe508del and KO rat ALIs had significantly diminished responses to the CFTR agonist forskolin, with KO rats more severely affected. Similarly, ALI cultures derived from CF G542X rat tracheal cells were previously found to have significantly reduced forskolin-stimulated I_sc_ when compared to wild-type [6]. Studies conducted on excised CF rat tracheal tissue also demonstrate reduced CFTR function [6, 29]. Comparatively, studies characterising CFTR ion channel function in excised CF mouse tracheal tissue demonstrate a significant chloride secretory response to forskolin. This finding has been attributed to the presence of alternative chloride secretory pathways that are more dominant in murine airways and compensate for the lack of CFTR function [30]. The presence of defective CFTR-mediated chloride secretion in the tracheal airways of CF rat models suggests that rats are superior to mice when studying aspects related to airway physiology.

Phe508del rat ALI cultures were found to be responsive to the modulator combination ETI, with treated cells demonstrating a forskolin response that was restored to 59% of the wild-type level. In a previous study conducted on Phe508del rat nasal-derived ALIs treated with lumacaftor-ivacaftor, the forskolin response was similarly improved to 47% of wild-type level [4]. In comparison, the improvements in CFTR activity in human cells appear to be more substantial. One study conducted on nasal brushing-derived ALIs from CF patients with Phe508del/unknown variants showed that ETI restored the forskolin response to 83% of the non-CF level [31].

The lower-level CFTR restoration observed in modulator-treated Phe508del CF rat airway cells both here and previously [4] may be attributed to differences in the rat CFTR protein structure when compared to humans. Previous studies have shown that species-dependent differences in the CFTR protein can alter the response to CFTR modulator drugs. Corrector molecules such as lumacaftor have been found to rescue misfolded mouse Phe508del-Cftr protein, whereas potentiator molecules such as ivacaftor completely fail to augment mouse Gly551Asp-Cftr or Phe508del-Cftr function [32, 33]. In contrast, these results suggest that rat Phe508del-CFTR is amenable to potentiation by ivacaftor, as an improved response to forskolin stimulation would not have otherwise been observed. While ETI did not completely restore CFTR function in the Phe508del rat ALI cultures, our validation of a beneficial effect underscores the utility of this CF rat model and has already enabled studies assessing phenotype normalisation following in vivo treatment with modulators [28].

Rat airway epithelial cells were successfully transduced with VSV-G pseudotyped LV-GFP vector at MOIs of 1, 10, and 100, resulting in dose-dependent increases in transduction efficacy. Rat basal cells transduced with LV-CFTR at an MOI of 1 demonstrated that as little as 1% corrected cells could significantly restore CFTR function. Others have shown similar restoration of CFTR function in CF human airway epithelial cells treated with LV-CFTR vector followed by differentiation at ALI [20]. In this study, we went on to demonstrate comparable levels of CFTR restoration with gene therapy and ETI treatment, with gene-addition producing 47% and 68% increase toward wild-type in KO and Phe508del respectively, and ETI achieving 59% improvement. Use of higher MOI such as 10 or 100 is likely to have resulted in further increases in CFTR function, however, higher MOIs are not expected to be achievable in vivo or in clinical situations, and therefore were not tested in this study.

Assessment of mRNA expression via qPCR confirmed the presence of h*CFTR* transcripts in the LV-treated rat ALI cultures, providing clear evidence that the improvements in ion transport were a direct result of the LV-CFTR gene-addition. The Phe508del-treated group had significantly higher h*CFTR* mRNA expression than the KO treated group, potentially suggesting that Phe508del cells are more transducible than KO cells, although further investigation is required to confirm this. The number of h*CFTR* transcripts in the treated samples were approximately ten times lower than the wild-type endogenous rat *CFTR* levels, suggesting only small amounts of wild-type *CFTR* mRNA are needed to significantly restore CFTR function. The levels of rat *CFTR* mRNA transcripts were not altered following treatment, indicating that LV-CFTR gene addition does not affect endogenous transcription.

One limitation of this study was that the basal cells were transduced with LV-CFTR rather than differentiated airway cultures, which is more representative of an in vivo setting and is likely to require a much higher MOI when compared to the MOI of 1 that was used here. However, there is value in this approach as the basis of an airway-directed stem cell therapy where basal cells are transduced ex vivo and then delivered into the airways [15]. Thus, determining the transduction efficiency of basal cells and the effects on differentiation of the epithelium are still of significant value. Another limitation is that we utilised the GFP reporter gene to determine which MOI to use for the LV-CFTR studies. Although we have no evidence to suggest that this is not a reliable approach, there is potential for some discrepancy in the expression levels between the two transgenes.

This investigation has laid the groundwork for future studies that will assess the in vivo effectiveness of LV-CFTR gene therapy in the lungs of CF rats. We have previously shown corrected CFTR function in the nasal airways of CF rats [18] and CF mice [17, 27] however, the target region of gene therapy for people with CF will be the small airways of the lungs as this is where the disease initiates. Future work will include treating the lungs of CF rats with gene therapy and assessing improvement in CFTR function via lower airway transepithelial potential difference or Ussing chamber measurements on excised airway tissue. Assessment of lung function with measures such as respiratory mechanics (e.g. flexiVent) or functional lung imaging (e.g. X-ray velocimetry) may also prove valuable in assessing the efficacy of LV vector-mediated gene therapy [34].

In summary, our findings highlight the potential of a LV vector-based gene therapy for the treatment of CF lung disease. We showed that Phe508del rat ALI cultures had improved CFTR ion transport following treatment with ETI, demonstrating that rat Phe508del-CFTR protein trafficking and gating can be rescued with this modulator combination. LV-CFTR treatment of Phe508del and KO rat cultures produced comparable CFTR function restoration to ETI, illustrating the effectiveness of this therapeutic approach. This work provides further support for the continued development of a gene-addition therapy for CF and offers the possibility to provide a mutation-agonistic treatment for those without modulator therapies.

## Supplementary information

Supplementary material

## Data Availability

Data will be made available upon request.
