# Supplementary material for: Lentiviral vector gene therapy and CFTR modulators show comparable effectiveness in cystic fibrosis rat airway models
Source: Gene Ther. 2024 Aug 25;31(11-12):553–9. doi: 10.1038/s41434-024-00480-y (PMC11576507; doi:10.1038/s41434-024-00480-y)
Supplement: Supplementary file 1 — Supplementary material [file 41434_2024_480_MOESM1_ESM.pdf]

Supplementary figures

Table S1: Primer sequences

**Primer**	**Sequence (5’ to 3’)**	**Product length (bp)**
Cyclophilin A (forward)	GCAGACATGGTCAACCCCACCG	96
Cyclophilin A (reverse)	TGGAACTTTGTCAAACAGCTCG	
Rat CFTR (forward)	AAGCTGAAAGCAGGTGGGAT	117
Rat CFTR (reverse)	TGCTCCGACCACAATGAACA	
LV-CFTR (forward)	GCGTCATCAAAGCATGCCAA	108
LV-CFTR (reverse)	GCGTCATCAAAGCATGCCAA	

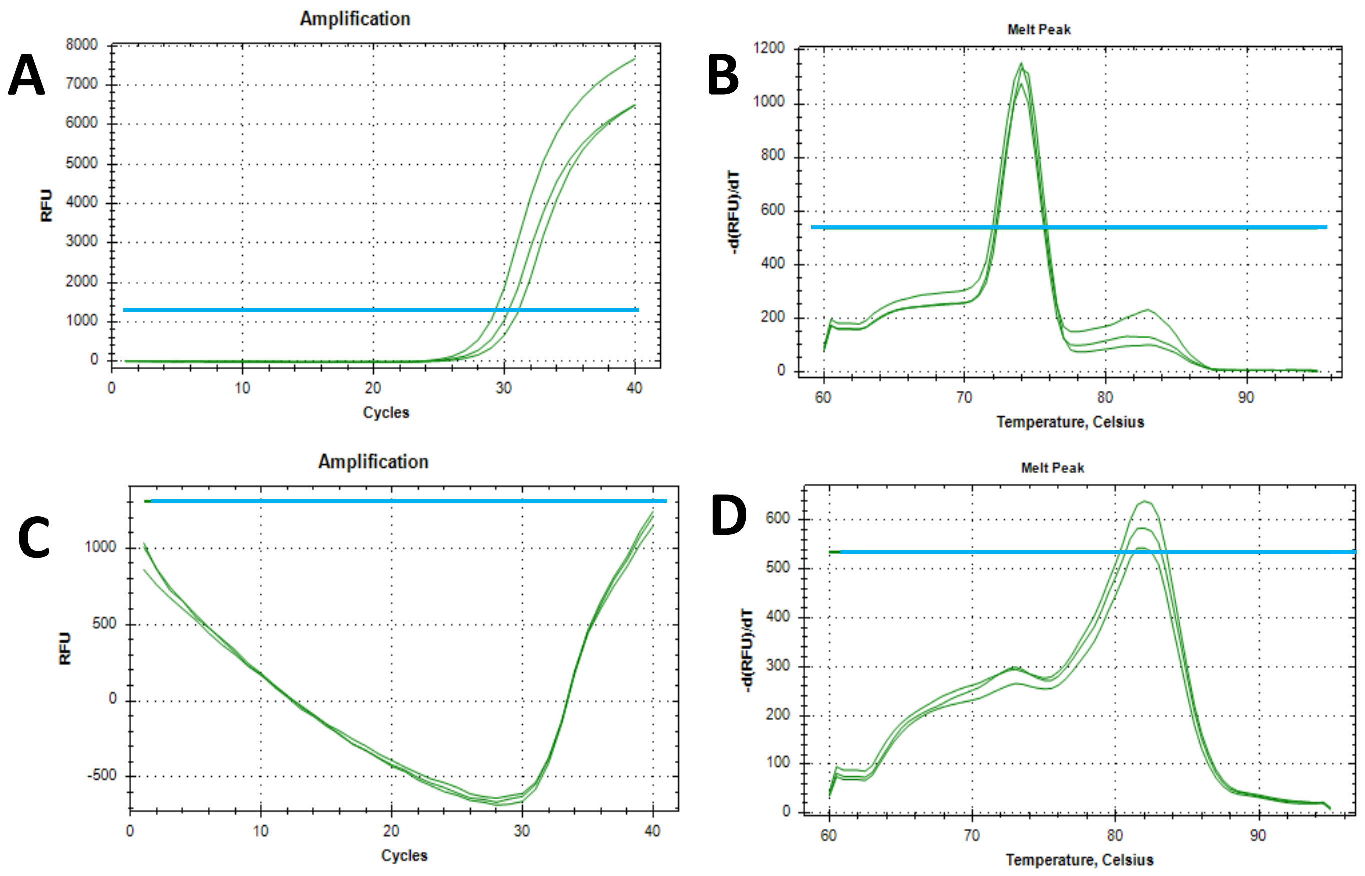

Figure S1: Specific primers were used to amplify rat *CFTR* transcripts in ALI culture samples. The amplification and corresponding melt curves show detection of rat *CFTR* transcripts in (**A and B**) rat ALI cultures, but not in (**C and D**) LV-*CFTR* plasmid samples, indicating the specificity of the primers for amplifying only rat *CFTR*. The blue line indicates the detection threshold.

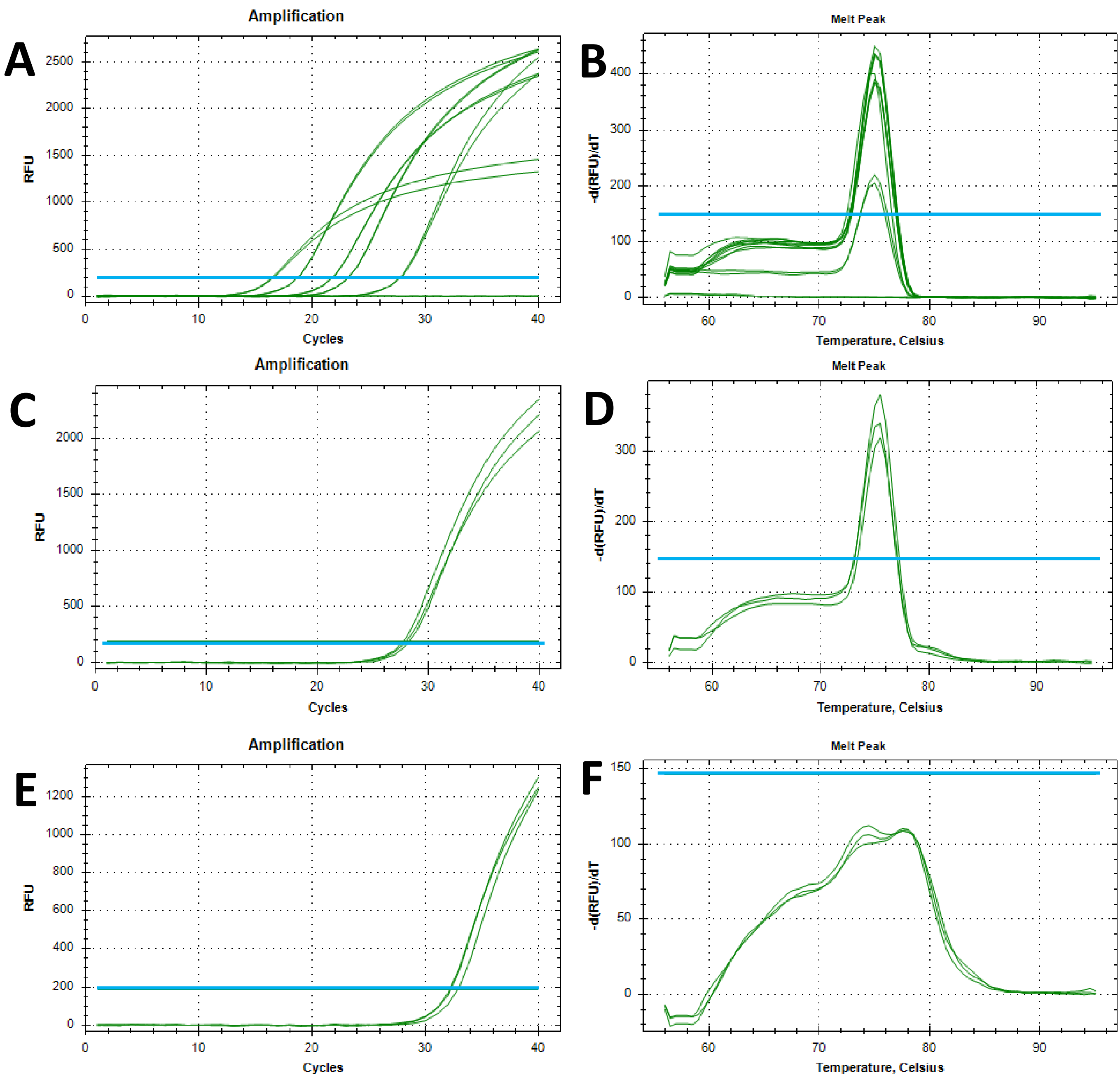

Figure S2: Specific primers were used to amplify human *CFTR* transcripts in ALI culture and LV-*CFTR* plasmid samples. The amplification and corresponding melt curves show detection of human *CFTR* in (**A** and **B**) LV-*CFTR* plasmid and (**C** and **D**) LV-*CFTR* treated ALI samples. There was no h*CFTR* detected in untreated ALI samples (**E** and **F**). The blue line indicates the detection threshold.

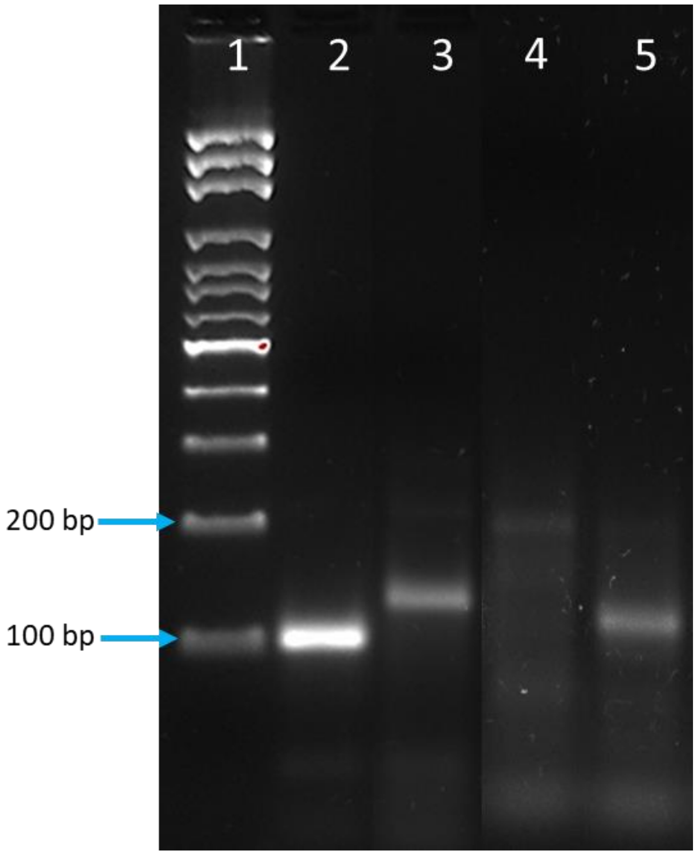

Figure S3: Gel electrophoresis of qPCR products demonstrates the specificity of human and rat CFTR primers. Lane 1 shows a 100 bp DNA ladder, lane 2 shows a 108 bp product corresponding to the amplification of h*CFTR* in a LV-*CFTR* plasmid sample, lane 3 shows a 117 bp product corresponding to the amplification of rat CFTR in a rat ALI sample, lane 4 shows no h*CFTR* product was amplified in an untreated rat ALI sample and lane 5 shows a 108 bp product demonstrating the presence of h*CFTR* in an LV-*CFTR* treated rat ALI sample.
